# Langanhaltende Krankheitskontrolle der disseminierten superfiziellen aktinischen Porokeratose (DSAP) mittels topischer Anwendung von 2% Simvastatin/2% Cholesterin‐Creme

**DOI:** 10.1111/ddg.15950_g

**Published:** 2026-07-07

**Authors:** Berenice M. Lang, Gregor Ojak, Mandy Crummenauer, Joanna Wegner, Caroline Mann, Stephan Grabbe, Petra Staubach

**Affiliations:** ^1^ Hautklinik und Poliklinik Universitätsmedizin Mainz; ^2^ Klinik für Dermatologie Universitätsmedizin Frankfurt am Main; ^3^ Dermatologisches Zentrum Dr. Lorenz und Koll, Kaiserslautern

**Keywords:** Hautkrebsprävention, Krankheitskontrolle, Porokeratose, topische Therapie, *unmet medical need*, zielgerichtete Therapie, Disease control, porokeratosis, skin cancer prevention, targeted therapy, topical therapy, unmet medical need

## Abstract

**Einleitung:**

Die disseminierte superfizielle aktinische Porokeratose (DSAP) ist eine seltene Keratinisierungsstörung, die mit einem erhöhten Risiko für kutane Tumoren verbunden ist.

**Zielsetzung:**

Ziel der Studie war die Entwicklung eines praktikablen und einfach anwendbaren Behandlungsregimes für die langfristige Kontrolle der DSAP einschließlich der Prävention neuer Hauttumoren.

**Patienten und Methodik:**

Insgesamt 19 Patienten mit DSAP wurden über einen Zeitraum von maximal 18 Monaten topisch mit einer Creme mit 2% Simvastatin und 2% Cholesterin (SC) behandelt. Der Schweregrad der Erkrankung wurde mittels *Investigator Global Assessment* (IGA) bewertet, während die Lebensqualität mittels *Dermatology Life Quality Index* (DLQI) erfasst wurde. Nebenwirkungen sowie die Entwicklung neuer Tumoren wurden dokumentiert.

**Ergebnisse:**

In allen behandelten Bereichen zeigte sich eine signifikante Verbesserung der IGA‐Werte (p < 0,001), wobei die meisten Fortschritte innerhalb der ersten 3 Monate auftraten und unter reduzierter proaktiver Anwendung zweimal wöchentlich erhalten blieben. Die DLQI‐Werte sanken innerhalb der ersten 3 Monate signifikant. Während der Behandlung traten keine neuen Hauttumoren auf.

**Schlussfolgerungen:**

SC‐Creme ist eine vielversprechende, zielgerichtete Langzeittherapie für DSAP, die nachhaltige Wirksamkeit, gute Verträglichkeit sowie verbesserte Lebensqualität bietet. Die individuelle Reduktion der Anwendung von täglicher zu proaktiver zweimal wöchentlicher Therapie könnte wesentlich für die Krankheitskontrolle sein und möglicherweise zu einer wichtigen Komponente bei der Prävention von Hauttumoren in dieser speziellen Patientengruppe werden.

## EINLEITUNG

Die disseminierte superfizielle aktinische Porokeratose (DSAP) ist eine seltene Keratinisierungsstörung der Haut, die sich durch genetische und klinische Heterogenität auszeichnet. Sie betrifft hauptsächlich sonnenexponierte Bereiche und manifestiert sich typischerweise mit asymptomatischen oder juckenden, rosafarbenen bis bräunlichen Papeln oder Plaques, umgeben von einem charakteristischen erhabenen Randsaum, der cornoiden Lamelle. Präzise epidemiologische Daten sind aufgrund der hohen Dunkelziffer und häufiger Fehldiagnosen begrenzt. Die Erkrankung tritt typischerweise zwischen dem vierten und fünften Lebensjahrzehnt auf, mit einer leichten Dominanz beim weiblichen Geschlecht. Es wurden sowohl autosomal‐dominante als auch sporadische Fälle beschrieben. Die disseminierte superfizielle aktinische Porokeratose tritt überwiegend bei Menschen mit heller Haut und chronischer Sonnenexposition auf. Neben ihrer kosmetischen und symptomatischen Belastung ist die DSAP mit einem erhöhten Risiko für Plattenepithelkarzinome verbunden, was die Bedeutung einer effektiven Langzeitkrankheitskontrolle unterstreicht. Verschiedene Quellen beziffern das Lebenszeitrisiko einer malignen Transformation auf 3% bis 11%, wobei das Risiko mit längerem Krankheitsverlauf steigt. Aktuelle Therapieoptionen, wie photodynamische Therapie, topisches Imiquimod, topisches 5‐Fluorouracil und topische wie systemische Retinoide, zeigen oft nur begrenzten Erfolg und gehen häufig mit Nebenwirkungen wie Irritationen, Erythem und Unwohlsein einher. Bisher existiert keine zugelassene, indikationsspezifische Therapie, was den hohen medizinischen Bedarf an wirksameren und gezielteren Behandlungsstrategien unterstreicht.^1^, ^2^

Aktuelle Fortschritte im Verständnis der Pathophysiologie der DSAP haben neue therapeutische Ansätze eröffnet. Die Erkrankung wurde mit Mutationen im Mevalonat‐Signalweg assoziiert, einem wichtigen Stoffwechselweg, der an der Cholesterinbiosynthese beteiligt ist. Diese Mutationen führen zur Anhäufung von Mevalonat und dessen Metaboliten, welche vermutlich zur entzündlichen Erscheinung der DSAP beitragen. Entsprechend ist die gezielte Beeinflussung des Mevalonat‐Signalwegs als vielversprechende therapeutische Strategie identifiziert worden.^1^, ^3^

Ein solcher Ansatz umfasst die topische Anwendung von Cholesterin und Statinen (zum Beispiel Lovastatin oder Simvastatin). Diese duale Therapie zielt darauf ab, Cholesterin – eine essenzielle Komponente der Hautbarriere – wiederherzustellen und gleichzeitig die Akkumulation toxischer Mevalonat‐Zwischenprodukte zu hemmen. Das Konzept wurde erstmals von Paller et al. im Jahr 2011 erfolgreich bei der Behandlung des kongenitalen Hemidysplasie‐Syndroms mit ichthyosiformer Erythrodermie und Extremitätendefekten (CHILD‐Syndrom) angewandt, einer verwandten Störung desselben Signalwegs.^4^ Im Jahr 2019 wurde dieser Ansatz von Atzmony et al. auf die DSAP übertragen und stellte einen entscheidenden Schritt in der therapeutischen Prüfung dar.^5^ Seitdem haben mehrere kleine Fallserien vielversprechende Ergebnisse gezeigt, einschließlich signifikanter Reduktionen von Läsionen, Erythem und Schuppung innerhalb weniger Wochen nach Anwendung.^6^, ^7^, ^8^, ^9^, ^10^ Eine kürzlich durchgeführte Metaanalyse mit 33 Patienten ergab eine Gesamtansprechrate von 93% nach zweimal täglicher Anwendung der Formulierung über acht Wochen.^11^ Daten zur Langzeitwirksamkeit und Sicherheit dieses Ansatzes sind jedoch noch begrenzt. Angesichts der chronischen Natur der DSAP und des Mangels an wirksamen Alternativen, stellt die Evaluation der langfristigen Krankheitskontrolle mittels einer Creme aus 2% Simvastatin und 2% Cholesterin (SC‐Creme) einen wesentlichen nächsten Schritt dar, um diesen Bedarf zu adressieren.

## ZIELSETZUNG

Ziel dieser Untersuchung war es, die Wirksamkeit und Sicherheit der topischen SC‐Creme in einer größeren Patientenkohorte über eine längere Behandlungsdauer zu prüfen. Unter Berücksichtigung der chronischen und progressiven Natur der DSAP wurde darüber hinaus angestrebt, eine strukturierte Langzeitmanagementstrategie zur nachhaltigen Krankheitskontrolle und Symptomstabilisierung zu entwickeln. Ferner wurde ein optimiertes Therapieschema vorgeschlagen, das Therapieadhärenz und langfristige Behandlungsergebnisse verbessern soll. Ein weiteres Ziel dieser Untersuchung war die Bewertung, ob die SC‐Therapie bei der Prävention der malignen Transformation, insbesondere der Entwicklung von Plattenepithelkarzinomen bei DSAP‐Patienten, eine Rolle spielen könnte. Um das derzeit begrenzte Verständnis der Krankheitslast besser zu erfassen, wurde zudem die gesundheitsbezogene Lebensqualität (QoL) mittels Dermatology Life Quality Index (DLQI) bewertet, da solche Daten bislang nicht berichtet wurden.

## PATIENTEN UND METHODIK

Alle Patienten mit der Diagnose DSAP, die zwischen März 2022 und Juni 2023 an der Hautklinik der Universitätsmedizin Mainz behandelt wurden, wurden für diese Analyse auf ihre Eignung geprüft. Der Nachbeobachtungszeitraum betrug bis zu 18 Monate. Die Behandlung erfolgte im Rahmen der klinischen Routineversorgung und alle Patienten wurden über das individualisierte therapeutische Vorgehen informiert. Die SC‐Creme wurde als Magistralrezeptur von örtlichen Apotheken mit folgender Zusammensetzung hergestellt: 2% Simvastatin, 2% Cholesterin, konserviertes Wasser, *Unguentum* c*ordes*. Das empfohlene Behandlungsprotokoll sah eine zweimal tägliche Applikation der SC‐Creme auf die betroffenen Bereiche vor. Nach bestätigter guter Verträglichkeit und initialem klinischen Ansprechen wurde die Anwendungshäufigkeit auf einmal täglich reduziert (typischerweise nach 8–12 Wochen). Langfristig wurde eine proaktive Erhaltungstherapie eingeführt, bei der die Creme in der Regel zweimal wöchentlich aufgetragen wurde, meist innerhalb der ersten sechs Monate nach Behandlungsbeginn.

Patienten wurden eingeschlossen, wenn sie mindestens einen Nachuntersuchungstermin wahrnahmen und eine Fotodokumentation zu Studienbeginn sowie mindestens einem weiteren Zeitpunkt vorlag. Klinische Fotografien erfolgten zu Beginn (Monat 0) sowie nach Möglichkeit nach 3, 6, 9, 12, 15 und 18 Monaten (± 1 Monat).

Die Schwere der Erkrankung wurde durch zwei Ärzte mittels eines fünfstufigen *Investigator Global Assessment* (IGA) bewertet (0 = keine Läsionen, 1 = gering, 2 = leicht, 3 = moderat, 4 = schwer, 5 = sehr schwer). Die Daten wurden anonymisiert ausgewertet. Patienten füllten zusätzlich den DLQI aus, um die QoL zu erfassen. Neu auftretende kutane Tumoren sowie therapieassoziierte Nebenwirkungen wurden dokumentiert. Demographische Merkmale, einschließlich Alter bei Therapiebeginn, Geschlecht, frühere Behandlungen und Hautkrebsanamnese wurden erfasst.

Die statistische Analyse erfolgte mittels deskriptiver Methoden. Absolute und relative Häufigkeiten wurden für kategoriale Variablen berechnet. Aufgrund der kleinen Stichprobengröße wurde die Signifikanz der DLQI‐Werte mit dem Wilcoxon‐Test analysiert. Zur Bewertung klinischer Veränderungen über die Zeit wurden *Linear Mixed Models* (LMM) unter Nutzung von SPSS angewandt, um Korrelationen innerhalb der Individuen zu berücksichtigen. Getrennte LMM wurden für verschiedene anatomische Regionen durchgeführt. Die Schätzung erfolgte mittels residualer Maximum‐Likelihood‐Methode (REML), wobei fehlende Daten unter der Annahme *missing at random* (MAR) behandelt wurden.

## ERGEBNISSE

### Patientencharakteristika

Insgesamt 26 Patienten mit DSAP begannen im Beobachtungszeitraum eine Therapie mit SC‐Creme. Davon wurden 19 Patienten in die finale Analyse eingeschlossen; sieben Patienten mussten aufgrund unzureichender fotografischer Dokumentation zu Therapiebeginn ausgeschlossen werden, da eine klinische Bewertung nicht möglich war. Das mittlere Alter der Patienten bei Therapiebeginn lag bei 66,89 Jahren (Bereich: 45–85 Jahre). Die Mehrheit der Patienten war weiblich (58%). Vierzehn Patienten (74%) hatten bereits vorherige Therapien der DSAP erhalten, davon hatten elf Patienten (58%) mehr als einen Therapieansatz ausprobiert. Zu den am häufigsten genannten Vorbehandlungen gehörten topisches Imiquimod (5%‐ oder 3,75%‐Creme; 71%), photodynamische Therapie (Rotlicht und/oder [künstliches] Tageslicht; 64%) sowie topische Diclofenac‐Hyaluronsäure‐Creme (36%). Weitere Vortherapien umfassten topisches 5‐Fluorouracil, Kryochirurgie, topisches Ingenolmebutat, Peelings mit Alpha‐Hydroxysäuren, topisches Calcipotriol sowie topische und systemische Retinoide, jeweils in verschiedenen Dosierungsregimen (Tabelle 1). Die längste Nachbeobachtungszeit betrug in dieser Kohorte 18 Monate (n = 4, 21%). Sieben Patienten (37%) wurden nach 15 Monaten und acht Patienten (42%) nach 12 Monaten evaluiert.

**TABELLE 1:** Patientencharakteristika.

	n = 19
**Alter** [Jahre (Spanne)]	66,89 (45–85)
**Geschlecht** [n (%)]	
Männlich	8 (42)
Weiblich	11 (58)
**Vortherapien** [n (%)]	
Insgesamt	14 (74)
Mehr als eine	11 (58)
Imiquimod	10 (71)
Photodynamische Therapie	9 (47)
Diclofenac‐Hyaloronsäure	5 (36)
**Hautkrebs in der Anamnese [n (%)]**	
Insgesamt	4 (21)
Plattenepithelkarzinom	4 (21)
Basalzellkarzinom	4 (21)
Melanom	1 (5)
Aktinische Keratose	8 (42)
**Betroffene Körperregion [n (%)]**	
Linker Arm	16 (84)
Rechter Arm	16 (84)
Linkes Bein	14 (74)
Rechtes Bein	14 (74)
Brust	2 (11)
Abdomen	1 (5)

*Abk*.: HA, Hyaluronsäure; PDT, photodynamische Therapie

### Wirksamkeit der SC‐Creme

**ABBILDUNG 1:**
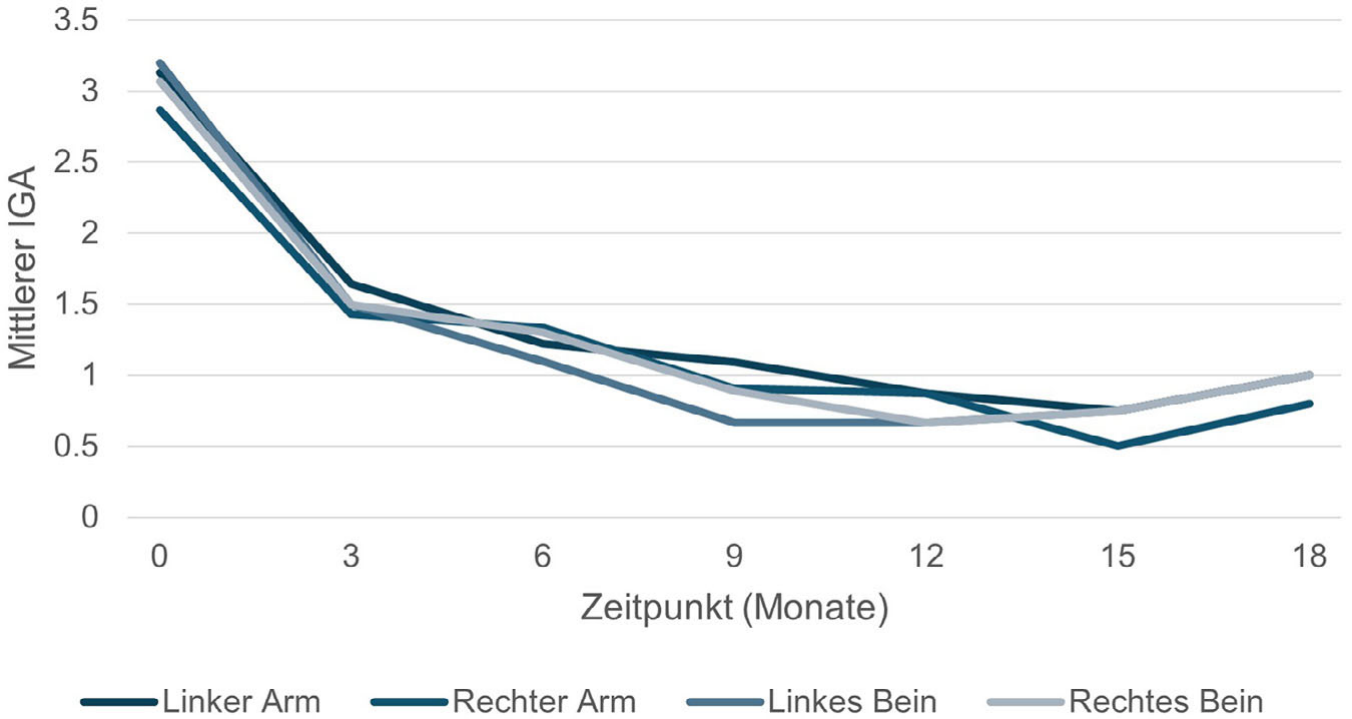
Durchschnittlicher IGA‐Wert für linken Arm, rechten Arm, linkes Bein und rechtes Bein zu verschiedenen Zeitpunkten der Analyse. Die IGA‐Reduktion war in allen untersuchten Körperregionen signifikant (p < 0,001).

Die am häufigsten betroffenen Körperregionen war die oberen Extremitäten (84%), gefolgt von den unteren Extremitäten (74%). Bei allen Patienten lagen bilaterale Läsionen vor. Zusätzlich hatten zwei Patienten (11%) eine Beteiligung des Dekolletés, und bei einem Patienten (5%) waren Läsionen am Abdomen vorhanden (Tabelle 1). Acht Patienten (42%) behandelten zwei Körperregionen, ein Patient (5%) drei Körperregionen, neun Patienten (47%) vier Körperregionen und zwei Patienten (11%) fünf Körperregionen. IGA‐Werte zeigten bei allen Patienten und behandelten Körperregionen eine Reduktion der Erkrankungsschwere (Tabelle 2, Abbildung 1). Es wurde eine signifikante Abnahme der IGA‐Werte im Zeitverlauf festgestellt, wobei die stärkste Verbesserung innerhalb der ersten 3 Monate der Behandlung auftrat. Dieser therapeutische Effekt blieb über den gesamten Beobachtungszeitraum von bis zu 18 Monaten erhalten. Die Verbesserung innerhalb der ersten 6 Monate war signifikant ausgeprägter als jene zwischen den Monaten 9 und 18. Um die Behandlungseffektivität im zeitlichen Verlauf zu bewerten, wurde eine LMM‐Analyse mit den Visitenzeitpunkten (Ausgangswert und alle 3 Monate bis 18 Monate) als fester Effekt durchgeführt. Zufällige Achsenabschnitte (random intercepts) für die einzelnen Patienten und zufällige Steigungen (random slopes) für den Faktor Zeit wurden berücksichtigt, um die individuelle Variabilität abzubilden. Eine autoregressive Kovarianzstruktur wurde verwendet, um die Korrelation der wiederholten Messungen zu modellieren. Die Ergebnisse zeigten signifikante Veränderungen im Verlauf für alle Extremitäten (rechter Arm: F(6; 31,949) = 16,361, p < 0,001; linker Arm: F(6; 31,890) = 19,527, p < 0,001; rechtes Bein: F(6; 31,109) = 27,949, p < 0,001; linkes Bein: F(6; 32,211) = 19,828, p < 0,001). Die geschätzten Randmittelwerte zeigten eine deutliche Reduktion der IGA‐Werte vom Ausgangswert (rechter Arm: M = 3,133, SE = 0,269; linker Arm: M = 2,867, SE = 0,247; rechtes Bein: M = 3,200, SE = 0,231; linkes Bein: M = 3,067, SE = 0,249) auf 18 Monate (rechter Arm: M = 0,748, SE = 0,377; linker Arm: M = 0,671, SE = 0,335; rechtes Bein: M = 0,853, SE = 0,337; linkes Bein: M = 0,818, SE = 0,390). Die Varianzen der zufälligen Achsenabschnitte für die Patienten betrugen für die Analyse des rechten Arms 0,649, des linken Arms 0,574, des rechten Beins 0,283 und des linken Beins 0,350, was auf eine geringe interindividuelle Variabilität zu Studienbeginn hinweist. Für Abdomen und Thorax konnte das Modell aufgrund unzureichender Datenpunkte nicht erstellt werden. Es wurden keine statistisch signifikanten Unterschiede in der Therapieantwort zwischen Armen und Beinen beobachtet. Für alle Extremitäten fielen die durchschnittlichen IGA‐Werte nach neunmonatiger Behandlung unter 1, was minimale oder keine klinischen Zeichen der DSAP bedeutet (Abbildung 1). Die durchschnittliche Reduktion der IGA‐Werte über alle Extremitäten betrug 1,55 Punkte nach 3 Monaten, 1,83 nach 6 Monaten und 2,18 nach 9 Monaten. Ab neun Monaten stieg die Variabilität der IGA‐Werte, was auf eine zunehmende Heterogenität der Therapieantwort bei geringer ausgeprägter Symptomausprägung hinweist. Eine vollständige Remission (IGA 0) aller behandelten Regionen erreichten drei Patienten (16%). Weitere zehn Patienten (53%) erzielten eine beste Antwort von IGA 1. Insgesamt waren 59% der Patienten nach der Behandlung entweder symptomfrei oder nur leicht betroffen. Es bleibt anzumerken, dass ab Monat 12 die verfügbaren Datenpunkte deutlich abnahmen, was die Robustheit der Analysen zu späteren Zeitpunkten einschränken könnte.

**TABELLE 2:** Durchschnittlicher *Investigator Global Assessment* (IGA)‐Wert für jede untersuchte Körperregion zu verschiedenen Zeitpunkten (in Monaten) der Analyse.

	Zeitpunkt (Monate)	0	3	6	9	12	15	18
Linker Arm	IGA	3,13	1,64	1,22	1,09	0,88	0,75	1,00
	n	15	14	9	11	8	4	5
Rechter Arm	IGA	2,87	1,43	1,33	0,91	0,88	0,50	0,80
	n	15	14	9	11	8	4	5
Linkes Bein	IGA	3,20	1,50	1,10	0,67	0,67	0,75	1,00
	n	15	14	10	9	6	4	5
Rechtes Bein	IGA	3,07	1,50	1,30	0,89	0,67	0,75	1,00
	n	15	14	10	9	6	4	4
Brust	IGA	2	1	0	–	1	0,5	0
	n	2	2	1	–	1	2	1
Abdomen	IGA	3	2	–	1	–	–	–
	n	1	1	–	1	–	–	–

Abk.: IGA, Investigator Global Assessment

**ABBILDUNG 2:**
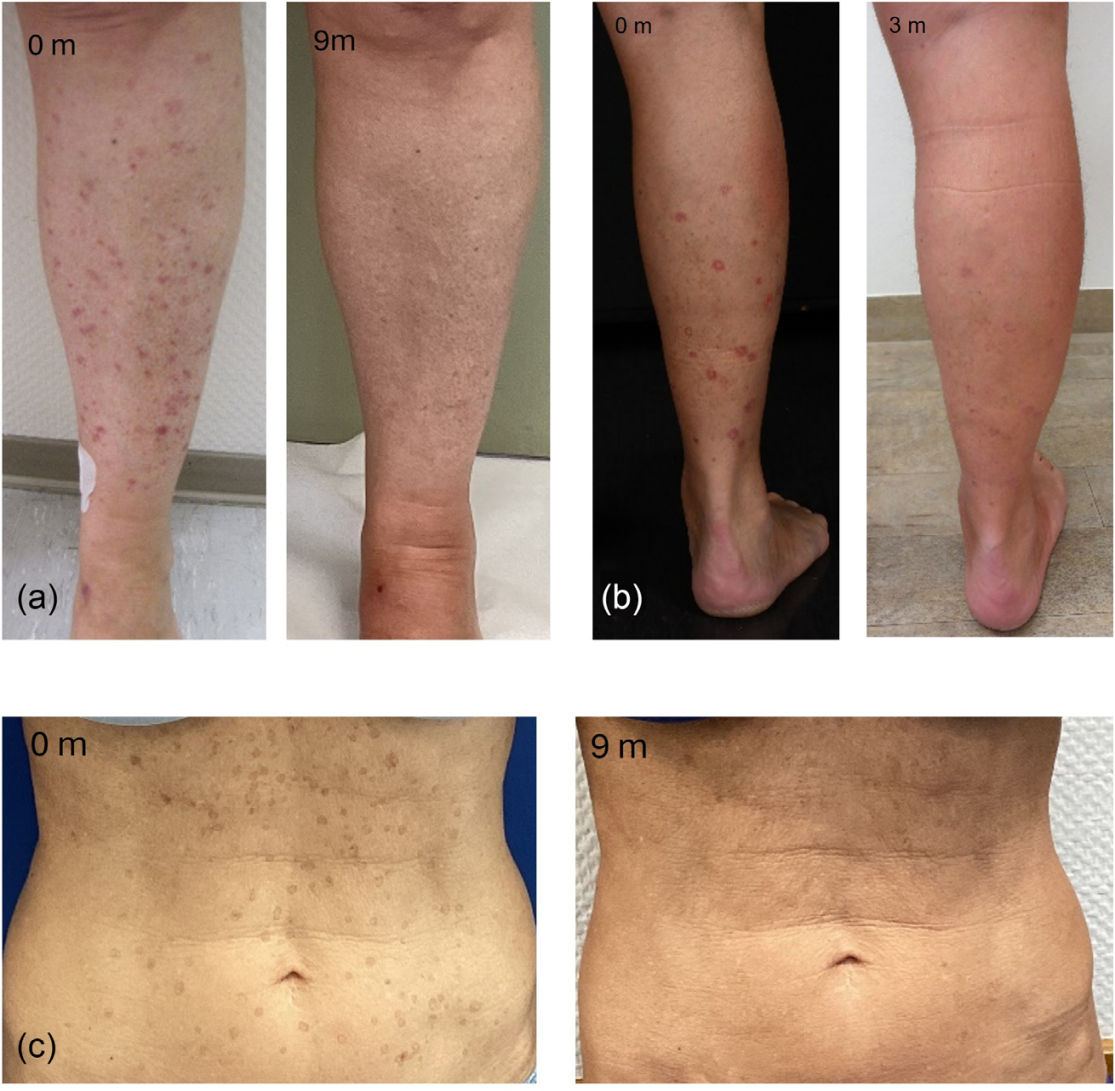
(a) Patient 8, linkes Bein; (b) Patient 10, rechtes Bein; (c) Patient 18, Abdomen. Zeitpunkte wie in der jeweiligen Fotografie angegeben.

Repräsentative klinische Fotografien von drei Patienten sind in Abbildung 2 dargestellt.

### Lebensqualität der Patienten

**ABBILDUNG 3:**
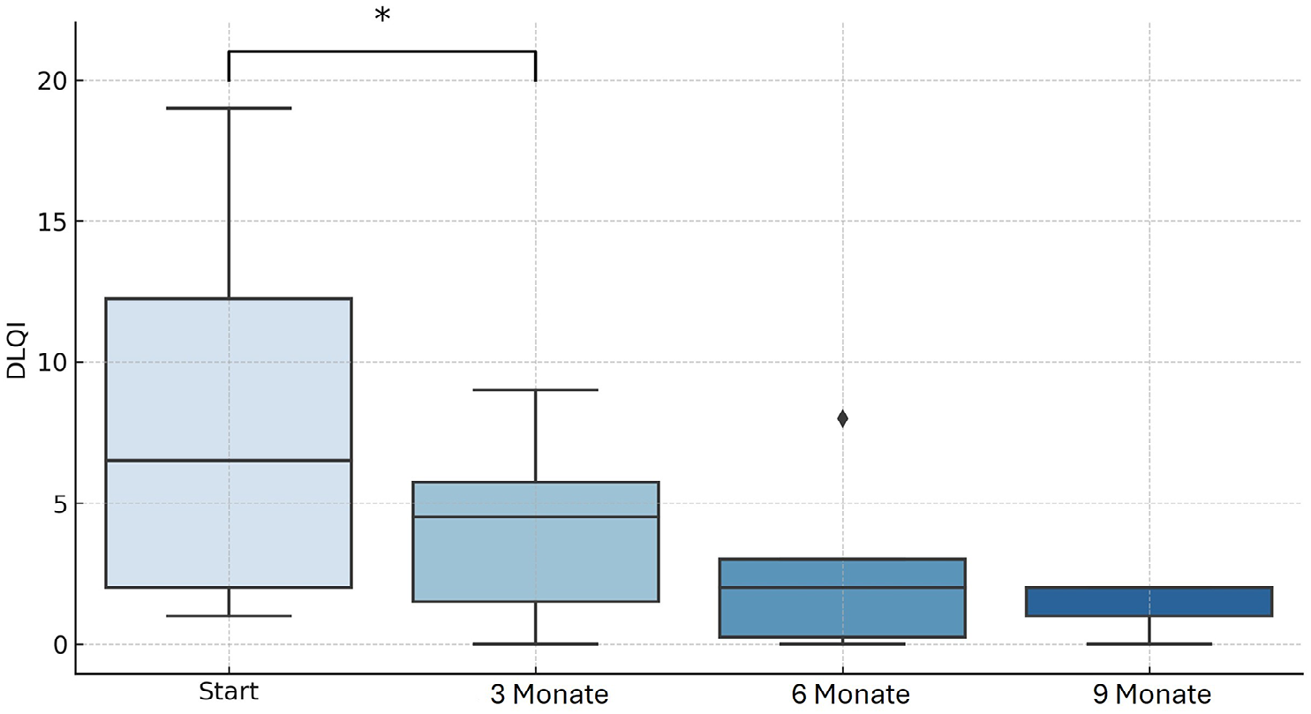
Mediane DLQI‐Werte zu den angegebenen Zeitpunkten. Statistisch signifikante Verbesserung im dritten Monat im Vergleich zum Ausgangswert (p = 0,027).

Die Auswirkungen der DSAP auf die QoL der Patienten wurden anhand des DLQI erfasst, der bei den Präsenzvisiten vor der klinischen Untersuchung ausgefüllt wurde. Aufgrund des Studiendesigns im Rahmen der klinischen Routineversorgung wurden die DLQI‐Daten nicht bei jedem Besuch systematisch erhoben. Insgesamt acht Patienten (42%) füllten den DLQI sowohl zu Beginn der Behandlung als auch mindestens einmal während der Therapie (nach 3, 6 und/oder 9 Monaten) aus. Die DLQI‐Werte zeigten im Zeitverlauf einen klar rückläufigen Trend, was auf eine kontinuierliche Verbesserung der gesundheitsbezogenen QoL hinweist. Zu Behandlungsbeginn gaben die Patienten eine moderate Beeinträchtigung ihrer QoL an (Median: 6,5; Mittelwert: 8,25), mit einem Wertebereich von 1 (keine Beeinträchtigung) bis 19 (sehr starke Beeinträchtigung). Nach 3 Monaten Therapie sank der Median‐DLQI‐Wert auf 4,5 (leichte Beeinträchtigung; Mittelwert: 3,67), was eine statistisch signifikante Verbesserung im Vergleich zum Ausgangswert darstellte (p = 0,027). Nach 6 Monaten sank der Median weiter auf 2,0, was der Schwelle für keine Beeinträchtigung nahekommt und einen Trend zur Signifikanz zeigte (p = 0,094). Im neunten Monat blieb der Median stabil bei 2,0, wobei der Unterschied zum Ausgangswert nicht mehr statistisch signifikant war (p = 0,25), was vermutlich auf die kleine Stichprobengröße zurückzuführen ist. Die Streuung der DLQI‐Werte war zu Beginn am höchsten und nahm im Verlauf ab, mit Wertebereichen zwischen 0 und 8 sowohl nach drei als auch nach 6 Monaten. Vergleiche zwischen späteren Zeitpunkten ergaben keine statistisch signifikanten Unterschiede, was darauf hindeutet, dass die wesentlichste Verbesserung der Lebensqualität in der Regel innerhalb der ersten 3 Monate der Behandlung erfolgt (Abbildung 3).

### Tumorentwicklung und Nebenwirkungen

Da DSAP als Risikofaktor für die Entwicklung von Hautkrebs – insbesondere für das kutane Plattenepithelkarzinom (Transformationsrisiko etwa 3 bis 11%)^1^ – gilt, lag ein besonderer Fokus dieser Untersuchung auf dem Auftreten neuer Tumorläsionen während der Behandlung mit SC‐Creme. Etwa ein Fünftel der Studienpopulation (n = 4; 21%) hatte anamnestisch bereits invasive Hauttumoren, darunter Plattenepithelkarzinome (n = 4; 21%), Basalzellkarzinome (n = 4; 21%) sowie ein malignes Melanom (n = 1; 5%). Zusätzlich wiesen acht Patienten (42%) eine dermatohistopathologisch bestätigte aktinische Keratose in der Vorgeschichte auf (Tabelle 1). Während der gesamten Beobachtungszeit wurden keine neuen malignen Hauttumoren bei Patienten unter Therapie mit SC‐Creme festgestellt.

Die SC‐Creme wurde insgesamt gut vertragen. Einzelne Patienten äußerten Unzufriedenheit mit der Konsistenz der Creme, die als zu fettig beschrieben wurde. Unerwünschte Ereignisse wurden bei drei Patienten (16%) dokumentiert, darunter zwei Fälle von ekzematösen Hautveränderungen und ein Fall von intermittierendem Pruritus. Alle drei betroffenen Patienten wiesen eine bekannte atopische Diathese auf. Bei einem Patienten wurde klinisch eine Typ‐IV‐Sensibilisierung gegen Bestandteile der SC‐Creme vermutet; ein Epikutantest zur Bestätigung wurde jedoch nicht durchgeführt, und die Behandlung wurde beendet. Ein weiterer Patient, der aufgrund fehlender fotografischer Ausgangsdokumentation nicht in die Wirksamkeitsanalyse eingeschlossen wurde, entwickelte eine bestätigte Typ‐IV‐Allergie gegen Simvastatin, die durch Epikutantestung nachgewiesen wurde. In diesem Fall erwies sich Atorvastatin als geeignete Alternative für die Rezeptur.

## DISKUSSION

Die disseminierte superfizielle aktinische Porokeratose ist eine chronische, therapieresistente Hauterkrankung mit einem erheblichen ungedeckten medizinischen Bedarf. Obwohl zahlreiche therapeutische Optionen existieren, zeigen diese keine durchgehend überzeugende Wirksamkeit und sind häufig mit beträchtlichen Nebenwirkungen verbunden. Zudem ist DSAP mit einem erhöhten Risiko für epitheliale Hauttumoren, insbesondere dem Plattenepithelkarzinom, assoziiert. Angesichts der chronischen Natur der Erkrankung und ihres onkogenen Potenzials sind effektive Langzeittherapiestrategien von hoher klinischer Relevanz.

Das wachsende Verständnis der molekularen Pathogenese von DSAP, insbesondere der Zusammenhang mit Mutationen im Mevalonat‐Stoffwechselweg, untermauert das Rationale für zielgerichtete Therapien. Topische Formulierungen, die Statine und Cholesterin kombinieren, stellen einen neuartigen Therapieansatz dar, der die zugrunde liegende metabolische Dysfunktion direkt adressiert. Während in unserer Studie Simvastatin verwendet wurde, zeigen frühere Arbeiten auch die Anwendbarkeit von Lovastatin oder Atorvastatin in vergleichbaren Magistralrezepturen und belegen damit die Übertragbarkeit und das Potenzial dieses Ansatzes.^5^, ^7^, ^9^, ^10^

Jedoch ist die bisherige Literatur zu Statin‐/Cholesterin‐basierten Therapien begrenzt und umfasst meist Fallserien mit weniger als einem Dutzend Patienten und kurzen Beobachtungszeiträumen von acht bis zwölf Wochen.^5^, ^6^, ^7^, ^8^, ^9^, ^10^ Angesichts des chronischen Verlaufs von DSAP ist jedoch eine langfristige Krankheitskontrolle essenziell. Unsere Analyse erweitert dieses Wissen durch einen längeren Beobachtungszeitraum von bis zu 18 Monaten und liefert somit wertvolle Erkenntnisse zur anhaltenden Wirksamkeit der SC‐Creme.

Die Charakteristika unserer Kohorte stimmen mit der bisherigen Literatur überein: Die Patienten waren im mittleren Lebensalter (Durchschnitt 66,89 Jahre), zeigten eine leichte weibliche Überrepräsentation und hatten bereits mehrere erfolglose Vorbehandlungen durchlaufen. Die Läsionen traten vorrangig an den bilateral betroffenen Extremitäten auf – ein klinisches Bild, das typisch für DSAP ist. In Übereinstimmung mit bisherigen Berichten zeigte unsere Kohorte eine sehr hohe therapeutische Ansprechrate auf die SC‐Creme. Eine erste Metaanalyse zur Statintherapie bei DSAP mit 33 Patienten ergab, dass über 90% der Patienten eine klinische Verbesserung der Hautläsionen erfuhren – bei durchgehender zweimal täglicher Anwendung über 8 Wochen.^11^ Auch in unserer Studie konnten signifikante IGA‐Verbesserungen innerhalb der ersten 3 Monate beobachtet werden. Unser Schwerpunkt lag jedoch auf dem Erhalt des therapeutischen Effekts bei gleichzeitig reduzierter Applikationsfrequenz, um die langfristige Compliance und Krankheitskontrolle zu fördern. Ein zentrales Anliegen bei einer längerfristigen topischen Therapie ist die Patientenadhärenz, insbesondere bei häufig erforderlicher Anwendung. Deshalb entwickelten wir ein proaktives Therapieschema mit schrittweiser Reduktion der Applikationsfrequenz bis hin zu einer zweimal wöchentlichen Erhaltungsbehandlung. Dieser Ansatz – inspiriert von Behandlungsstrategien bei anderen chronisch‐entzündlichen Dermatosen wie der atopischen Dermatitis – könnte die Therapietreue verbessern und gleichzeitig die Krankheitskontrolle sichern.^12^, ^13^, ^14^ Unsere Ergebnisse zeigen, dass der Großteil der therapeutischen Wirkung innerhalb der ersten sechs Monate erzielt wird und bei reduzierter Anwendungshäufigkeit über den weiteren Verlauf erhalten bleibt, was die Effektivität des proaktiven Ansatzes bei DSAP unterstützt.

Bemerkenswert war die einheitliche Therapieantwort über alle untersuchten Extremitäten hinweg, ohne signifikante Unterschiede zwischen Armen und Beinen. Dies ist insofern relevant, als chronisch‐entzündliche Hauterkrankungen häufig an den unteren Extremitäten eine langsamere Rückbildung zeigen.^15^, ^16^, ^17^ Das gleichmäßige Ansprechen bei DSAP deutet darauf hin, dass die anatomische Lokalisation eine untergeordnete Rolle im Behandlungsverlauf spielt.

Die Daten zur QoL bei DSAP‐Patienten sind nach wie vor begrenzt. Blyth et al. berichteten über einen Ausgangs‐DLQI‐Median von 5 (Bereich: 2–21), erhoben jedoch keine weiteren Werte im Verlauf oder nach der Behandlung.^6^ In unserer Kohorte zeigte sich ein DLQI‐Ausgangsmedian von 6,5 (Bereich: 1–19), was auf eine moderate Beeinträchtigung mit hoher interindividueller Variabilität hinweist – ein Ausdruck der heterogenen Krankheitslast. Interessanterweise korrelierte der Ausgangs‐DLQI nicht mit dem klinisch erfassten Schweregrad (IGA). Aufgrund der Datenerfassung innerhalb der Regelversorgung wurde der DLQI nicht systematisch erhoben. Dennoch zeigen die verfügbaren Daten eine signifikante Verbesserung der Lebensqualität innerhalb der ersten drei Monate, die sich über ein Jahr hinweg aufrechterhalten ließ. Weitere Studien mit größeren Patientenkollektiven sind notwendig, um dieses wichtige Thema näher zu untersuchen.

Die SC‐Creme wurde insgesamt gut vertragen. Unerwünschte Ereignisse waren auf drei Patienten (16%) beschränkt, bei denen ekzematöse Läsionen oder intermittierender Pruritus auftraten – Symptome, die möglicherweise einer atopischen Diathese zugrunde liegen. In einem Fall wurde eine Typ‐IV‐Sensibilisierung vermutet, jedoch mangels Epikutantestung nicht bestätigt. Bemerkenswerterweise dokumentierten wir einen bestätigten Fall einer Typ‐IV‐Allergie gegen Simvastatin bei einem Patienten außerhalb dieser Kohorte – im Einklang mit einem früheren Bericht von Ahrens et al.^18^, ^19^ Als therapeutische Alternative wurde Atorvastatin gut vertragen, und eine systemische Einnahme von Simvastatin blieb weiterhin möglich. Ob die Sensibilisierung dem Wirkstoff selbst oder den Hilfsstoffen der Rezeptur zuzuschreiben ist, bleibt unklar und sollte angesichts der weit verbreiteten Off‐Label‐Herstellung von Statin‐Cremes aus oralen Darreichungsformen weiter untersucht werden.

Unsere Ergebnisse stützen die Anwendung der SC‐Creme als vielversprechende, personalisierte und zielgerichtete Langzeittherapieoption bei DSAP. Patienten zeigten eine ausgeprägte und anhaltende Verbesserung der Läsionsschwere – unabhängig vom Ausgangsschweregrad – bei insgesamt guter Verträglichkeit. Wichtig ist, dass es während der gesamten Behandlungsdauer nicht zu einer Neuentstehung von malignen Hauttumoren kam. Auch wenn daraus kein sicherer protektiver Effekt abgeleitet werden kann, legt es nahe, dass eine verbesserte Krankheitskontrolle möglicherweise zu einem reduzierten Risiko einer malignen Transformation beiträgt. Nach unserem Kenntnisstand handelt es sich um die erste Studie, die den langfristigen Einsatz der SC‐Creme mit schrittweiser Reduktion der Applikationshäufigkeit untersucht und deren Potenzial als tragfähige Erhaltungsstrategie bei chronischer DSAP unterstreicht.

### Limitationen

Mehrere Limitationen sind bei der Interpretation unserer Ergebnisse zu berücksichtigen. Die Untersuchung wurde in einem versorgungsnahen, klinischen Umfeld durchgeführt und nicht als kontrollierte Interventionsstudie, was zu einer gewissen Heterogenität in der Datenerhebung und in den Follow‐up‐Intervallen führte. Darüber hinaus wurde die Therapietreue nicht systematisch erfasst, was eine objektive Bewertung der Adhärenz einschränkt. Auch wenn der Beobachtungszeitraum von bis zu 18 Monaten im Vergleich zu bisherigen Studien deutlich verlängert wurde, reicht er nicht aus, um belastbare Aussagen zur tumorschützenden Wirkung der SC‐Creme zu treffen. Zukünftige Langzeitstudien mit standardisiertem Follow‐up und größeren Fallzahlen sind erforderlich, um diese vorläufigen Erkenntnisse zu validieren und zu erweitern.

### Schlussfolgerungen

Die topische Anwendung von SC‐Creme stellt einen bedeutenden Schritt hin zu einem zielgerichteten, individualisierten und patientenzentrierten Behandlungsansatz bei DSAP dar. Diese Studie zeigt das Potenzial der Formulierung auf, Krankheitsaktivität über einen längeren Zeitraum wirksam zu kontrollieren – bei gleichzeitig günstiger Verträglichkeit und Sicherheit. Künftige Forschung sollte darauf abzielen, Formulierungsbestandteile zu optimieren, Langzeitergebnisse umfassender zu evaluieren und den potenziellen Nutzen der Mevalonatweg‐Hemmung im Hinblick auf das Hautkrebsrisiko bei DSAP‐Patienten zu untersuchen. Darüber hinaus könnten standardisierte, kommerziell erhältliche Präparate sowie die Erforschung systemischer Applikationsformen die Zugänglichkeit und Wirksamkeit der Therapie weiter verbessern. Trotz des bestehenden Forschungsbedarfs zeigt sich die SC‐Creme bereits heute als vielversprechende Option für eine nachhaltige Langzeitkontrolle der DSAP.

## DANKSAGUNG

Open access Veröffentlichung ermöglicht und organisiert durch Projekt DEAL.

## INTERESSENKONFLIKT

Keiner.
